# Authentication of Herbal Supplements Using Next-Generation Sequencing

**DOI:** 10.1371/journal.pone.0156426

**Published:** 2016-05-26

**Authors:** Natalia V. Ivanova, Maria L. Kuzmina, Thomas W. A. Braukmann, Alex V. Borisenko, Evgeny V. Zakharov

**Affiliations:** Centre for Biodiversity Genomics, Biodiversity Institute of Ontario, University of Guelph, Guelph, Ontario, Canada; Cairo University, EGYPT

## Abstract

**Background:**

DNA-based testing has been gaining acceptance as a tool for authentication of a wide range of food products; however, its applicability for testing of herbal supplements remains contentious.

**Methods:**

We utilized Sanger and Next-Generation Sequencing (NGS) for taxonomic authentication of fifteen herbal supplements representing three different producers from five medicinal plants: *Echinacea purpurea*, *Valeriana officinalis*, *Ginkgo biloba*, *Hypericum perforatum* and *Trigonella foenum-graecum*. Experimental design included three modifications of DNA extraction, two lysate dilutions, Internal Amplification Control, and multiple negative controls to exclude background contamination. *Ginkgo* supplements were also analyzed using HPLC-MS for the presence of active medicinal components.

**Results:**

All supplements yielded DNA from multiple species, rendering Sanger sequencing results for *rbcL* and *ITS2* regions either uninterpretable or non-reproducible between the experimental replicates. Overall, DNA from the manufacturer-listed medicinal plants was successfully detected in seven out of eight dry herb form supplements; however, low or poor DNA recovery due to degradation was observed in most plant extracts (none detected by Sanger; three out of seven–by NGS). NGS also revealed a diverse community of fungi, known to be associated with live plant material and/or the fermentation process used in the production of plant extracts. HPLC-MS testing demonstrated that *Ginkgo* supplements with degraded DNA contained ten key medicinal components.

**Conclusion:**

Quality control of herbal supplements should utilize a synergetic approach targeting both DNA and bioactive components, especially for standardized extracts with degraded DNA. The NGS workflow developed in this study enables reliable detection of plant and fungal DNA and can be utilized by manufacturers for quality assurance of raw plant materials, contamination control during the production process, and the final product. Interpretation of results should involve an interdisciplinary approach taking into account the processes involved in production of herbal supplements, as well as biocomplexity of plant-plant and plant-fungal biological interactions.

## Introduction

Natural Health Products (NHP’s) are naturally-derived compounds used in naturopathic, homeopathic and traditional (e.g., Chinese) medicines [1]. Also known as nutraceuticals, they encompass a broad range of categories, from vitamins, minerals, and supplements to probiotics and herbal remedies. The latter are usually defined as products of plant origin, claimed to possess healing, rejuvenation properties or other positive health effects. The global annual market for herbal remedies, estimated at $83 billion [2], is becoming increasingly lucrative, while remaining less regulated, compared to pharmaceutical products.

Regulations concerning the composition of herbal remedies and allowable claims about their medicinal properties vary among countries [3]. Established industry standards for quality control are usually designed to detect harmful contaminants (e.g., arsenic) and to authenticate the presence of known biologically active (medicinal) components in the final product. Analyses are done using High Performance Liquid Chromatography (HPLC) [4,5], which, despite its wide acceptance, has a number of limitations. Firstly, it requires chemical references which are often expensive or unavailable for many biologically active components [6]. Secondly, HPLC test results are sensitive to variations in the manufacturing process (e.g., production methods), type of plant tissue used and even the geographic origin of plants [4,7,8]. Finally, standardization of chromatographic fingerprints used as diagnostic reference is inherently difficult; and there are no universally accepted industry standards [4]. The most concerning shortcoming of HPLC is its inability to detect adulteration of products, resulting in overlooked cases of substitution that have adversely impacted consumer health [9]. Adulteration often involves substitution of the source plant with another species which is cheaper but may possess similar biochemical properties. As a result, attention has been drawn to the need for improving herbal supplement authentication by introducing biological diagnostic methods, in addition to standard chemical testing [10,11].

DNA barcoding is a validated DNA-based approach providing species-level resolution that has received increasing acceptance as a regulatory tool for authenticating taxonomic provenance of commercial animal products, such as fish, meat and seafood [12–18]. Although standard DNA barcode markers have also been developed for plants [19,20] the outcome of initial research using this approach to authenticate herbal supplements [21–23] has been controversial. The findings of these studies motivated the New York attorney general to request DNA-based testing of herbal supplements from several major retailers; these tests failed to recover DNA from key listed medicinal plant species. The results of this inquiry led to the issue of cease-and-desist orders to prevent distribution of purportedly false-labeled herbal supplement products in the USA [24]. This move has been deemed premature [25], on the grounds that it disregarded the potential limitations of DNA sequencing techniques in detecting DNA in heavily processed plant extracts and herbal products containing more than one species (e.g. filler), as well as potential biases stemming from the laboratory procedures used to perform the testing and the resulting data interpretation. Most recent studies on DNA-based taxonomic authentication of herbal supplements [21,23,26] utilize Sanger sequencing which is negatively affected by primer bias, even when multiple extractions and serial dilutions are used to reduce preferential amplification of a non-target template, especially when DNA of the target species is degraded.

DNA degradation may happen during the production of standardized extracts. This process involves multiple stages [8] and uses a series of chemical and/or physical treatments to isolate the secondary metabolites of a medicinal plant, while selectively removing toxic or unwanted substances from the final products [27]. In addition to these methods, solid-state fermentation with various microorganisms is often used to improve the yield of active medicinal compounds [28–31]. These treatments often result in partial or complete degradation of plant DNA and often lead to contamination from foreign DNA sources [25].

An additional challenge for identification of DNA origin in plant materials is due to ecological interactions among species. Plants interact with a wide variety of organisms, including bacteria, viruses and fungi. Among these, plant-fungal relationships have key impact on plants through complex symbiotic, parasitic and pathogenic interactions [32]. Many potentially toxigenic fungi co-exist with plants as important endophytic and/or mycorrhizal symbionts [33–37]. A study of incidence and toxigenic capacity of fungi in Argentinian medicinal herbs [38] highlighted the need for standard procedures to assess acceptability limits for fungal contamination. As a result, DNA-based authentication protocols for such products must be sensitive enough to detect DNA template in mixed samples with varying concentration and levels of degradation.

Next-Generation Sequencing (NGS) offers several key advantages over Sanger sequencing: massive parallelization of sequencing reactions, clonal separation of templates, regardless of their relative concentration, superior sensitivity, and faster turnaround time [39,40]. This makes NGS the preferred method for analyzing samples with varying levels of DNA degradation, derived from multiple species, containing fillers, or contaminants. NGS was shown to be an effective and cost-efficient way to authenticate highly processed Traditional Chinese Medicine (TCM) products and to assist in monitoring their compliance with legal codes and safety regulations [41]. Despite this, there are relatively few studies utilizing this approach in authenticating herbal supplements.

Two recent comprehensive reviews on DNA-based authentication of botanicals [42,43] highlighted NGS as a prospective way to verify listed ingredients in herbal medicines and to detect adulteration. In particular, Countinho Moraes et al. [43] indicated that targeted enrichment [44,45] and whole chloroplast sequencing [46–49] have great potential to resolve closely related plant species. However, the complexity of bioinformatics and laboratory workflows can limit their applicability for diagnostic applications. Both reviews concluded that amplicon metabarcoding (NGS-facilitated DNA barcoding) can become a standardized tool for authentication of herbal supplements.

Considering the above mentioned advantages of NGS and the potential limitations of Sanger sequencing, there is urgency in establishing high-resolution standard NGS workflow with a simple bioinformatics pipeline for DNA-based taxonomic authentication of NHP’s, complementary to existing quality control procedures.

Our study aimed to provide the first comprehensive evaluation of the performance of NGS-based DNA barcoding in authenticating the taxonomic provenance of NHP’s, using select herbal supplements as an example. Specific goals were to:

1. develop a standardized NGS-based DNA barcoding approach for the assessment of herbal supplements and other plant-derived NHP’s;
2. compare the relative efficiency and reliability of NGS *vs*. Sanger sequencing when authenticating NHP’s, particularly, heavily processed herbal supplements with degraded DNA;
3. identify the methodological challenges of detecting and discerning the DNA of the source plant(s), filler ingredients and contaminants of herbal supplements;
4. characterize fungal species frequently found in common herbal supplements and their potential effects on the results DNA-based authentication;
5. evaluate the overall applicability of DNA-based diagnostic approaches and their complementarity with the existing industry standards for quality control and authentication of NHP’s, such as HPLC-MS.

## Material and Methods

### Material

To establish a reference barcode library for five medicinal plant species, we used herbarium specimens from the Royal Ontario Museum (TRT Green Plant Herbarium) and several freshly collected specimens which were deposited in the collection of the Biodiversity Institute of Ontario, University of Guelph. Voucher and sequence data for the corresponding specimens are available in the public BOLD dataset: [DS-RLMPCCDB]: http://dx.doi.org/10.5883/DS-RLMPCCDB.

Material was selected in a way to cover a wide range of morphological origins (dried aerial parts, seeds, and roots) and forms of preparation (plant extracts, extracts combined with raw plant material, material with and without fillers). Five species of medicinal plants were chosen for testing: *Echinacea purpurea*, *Valeriana officinalis*, *Ginkgo biloba*, *Hypericum perforatum* and *Trigonella foenum-graecum*. Fifteen herbal supplements, three from each of the above species from three different manufacturers, were purchased in local pharmacies and health food stores (Table 1). To minimise the number of factors that could contribute to PCR inhibition, only oil-free tablets or gelatine capsules were selected for testing.

**Table 1:** Summary of sequencing success for herbal supplements tested, arranged by species and, by form of preparation.

Scientific name/Supplement code	Preparation form (summary)	Listed plant fillers	Source DNA detected			Filler DNA detected		
			Sanger (*rbcL*)	Sanger (*ITS2*)	NGS (*ITS2*)	Sanger (*rbcL*)	Sanger (*ITS2*)	NGS (*ITS2*)
***Echinacea purpurea***								
Echinacea-1	Raw material (aerial parts)		**+**	**+**	**+**			
Echinacea-2	Extract	Starch, soy			**+**	**+**	**+**	**+**
Echinacea-3	Extract							
***Valeriana officinalis***								
Valeriana-4	Raw material (roots)		**+**		N/A			
Valeriana-5	Raw material (roots)		**+**		N/A			
Valeriana-6	Raw material (roots)				N/A			
***Ginkgo biloba***								
Ginkgo-7	Extract	Soy				**+**	**+**	**+**
Ginkgo-8	Extract							
Ginkgo-9	Raw material (leaves) + extract	Rice starch	**+**	N/A	**+**			
***Hypericum perforatum***								
Hypericum-10	Raw material (aerial parts)		**+**	**+**	**+**			
Hypericum-11	Extract	Rice flour			**+**			**+**
Hypericum-12	Extract	Rice powder			**+**			
***Trigonella foenum-graecum***								
Trigonella-13	Raw material (seeds)		**+**	**+**	**+**			
Trigonella-14	Raw material (seeds)		**+**	**+**	**+**			
Trigonella-15	Extract							

### DNA extraction

Prior to DNA extraction, each gelatine capsule was opened and the contents were transferred into 2 ml tubes (three capsules or tablets per supplement). Gloves were changed after each sampling event and special care was taken to decontaminate all working surfaces and tubes from the airborne powder using ELIMINase® (Decon Laboratories, Inc., King of Prussia, USA), DI water and ethanol.

According to the experiment setup, each of 15 supplements had six replicates per each of the three lysis buffers (three lysates and their corresponding 10× dilutions), resulting in a total of 18 replicates per supplement.

DNA extraction followed the previously published protocol for isolation of total genomic DNA from plants [50,51], with minor modifications to deal with powdered material, as described below. A volume equal to displacement of ~75–100 μl of fluid was transferred from the powdered material using pre-cut 1 ml sterile tips into a screw-top Matrix A grinding vial (MP Biomedicals, LLC, Santa Ana, USA) containing 1 ml of the corresponding lysis buffer: CTAB [52], ILB [53], or WHITL [54]. Tissue was ground in the presence of the lysis buffer at 28 Hz for 1 min using TissueLyzer II (Qiagen GmbH, Hilden, Germany) and tubes were incubated at 65°C for 2 hours. Following incubation, tubes were centrifuged for 1 min at 11,000×g. Lysates and their corresponding 10× dilutions were assembled into a 96-tube rack (12×8). 50 μl of each lysate were transferred to a new 96-well plate and extracted as described in [50,51] using a 1 ml Acroprep™ 96-well plate with 1 μm glass fiber media (Pall Life Sciences, Port Washington, USA) with two WB wash stages. DNA was eluted in 50 μl of 10 mM Tris-HCl pH 8.0. Each plate contained six blank wells filled with the corresponding lysis buffer used as an internal negative extraction control. Additionally, 50 μl of each of the three lysis buffers were dispensed into 32 wells of the 96-well plate and the entire plate was extracted as described above. This plate was used as a background contamination control.

### Internal amplification control

An Internal Amplification Control (IAC) was introduced to distinguish DNA degradation from PCR inhibition. To prepare the IAC plasmid, a 658 bp fragment of COI gene was amplified from lepidopteran DNA (*Crinodes ritsemae*) using LepF1-LepR1 [55] primers as described Hebert *et al*. [56]. The resulting product was cloned into the pCR™4-TOPO®Vector using the TOPO® TA Cloning Kit for Sequencing with One Shot® TOP10 Chemically Competent *E*. *coli* (Invitrogen, Thermo Fisher Scientific, Waltham, USA), according to manufacturer’s instructions. Resulting plasmid DNA was used as a positive internal control in PCR reactions. To find the optimal concentration of IAC, serial dilution (1 ng/μl, 0.1 ng/μl, 0.01 ng/μl, 0.001 ng/μl) was prepared from a 100 ng/μl control plasmid stock and amplified with LepF1-LepR1 [55] in the presence of 0.25 ng/ μl of *Brassica oleracea* DNA. We used the lowest IAC plasmid concentration that allowed the reliable amplification of both templates in a gradient of annealing temperatures from 45 to 56°C.

A short (~150 bp) region of *rbcL* was amplified in the presence of an IAC from supplements extracted with CTAB buffer. A volume of 1 μl from 0.1 ng/μl plasmid dilution and LepF1-LepR1 primers were added to a PCR master mix containing rbcLaF and MrbcL 163R primers (Table 2). The PCR followed standard protocols for plant *rbcL* amplification [51].

**Table 2:** Primers used for Sanger sequencing, NGS and in IAC.

Region/Primer name	Direction	Primer sequence	Reference
***rbcL***			
rbcLa-F	Forward	ATGTCACCACAAACAGAGACTAAAGC	[57]
rbcLa-R	Reverse	GTAAAATCAAGTCCACCRCG	[58]
MrbcL 163-R1	Reverse	CGGTCCAYACAGYBGTCCAKGTACC	this study
**IAC**			
LepF1	Forward	ATTCAACCAATCATAAAGATATTGG	[55]
LepR1	Reverse	TAAACTTCTGGATGTCCAAAAAATCA	[55]
***ITS2***			
ITS3	Forward	GCATCGATGAAGAACGCAGC	[59]
ITS_S2F	Forward	ATGCGATACTTGGTGTGAAT	[60]
ITS-S2F-GINK	Forward	ATGCGATATTTAGTGTGAAT	this study
ITS4	Reverse	TCCTCCGCTTATTGATATGC	[59]
**NGS-fusion**			
IonA	Forward	CCATCTCATCCCTGCGTGTCTCC[GACT][IonExpress-MID][**specific sequence**]	Ion Torrent, Thermo Fisher Scientific
trP1	Reverse	CCTCTCTATGGGCAGTCGGTGAT[**specific sequence**]	Ion Torrent, Thermo Fisher Scientific

### *rbcL*–Sanger sequencing workflow

The PCR with rbcLaF /rbcLaR primers [57,58] (Table 2) followed the protocol [51]. Bidirectional sequencing was done using the BigDye® Terminator v.3.1 Cycle Sequencing Kit (Applied Biosystems, Thermo Fisher Scientific) on an ABI 3730xl Genetic Analyzer (Applied Biosystems, Thermo Fisher Scientific) as described by [56,61]. Bidirectional sequences were assembled in CodonCode ver. 4.2.2 (CodonCode Corporation, Centerville, USA) and manually edited.

### *ITS2* –Sanger and NGS workflow

The PCR with ITS3/ITS_S2F/ITS4 primers [59,60] followed Fazekas et al. [51]. Thermocycling consisted of two rounds to minimize PCR bias which may be caused by fusion primers with MID tags [62]. The first round PCRs were performed at three annealing temperatures to minimize PCR bias further. The first round with regular (target) primers started with an initial denaturation at 94°C for 2 min, followed by 30 cycles of 94°C for 30 s, annealing at 51°C, 53°C and 56°C for 30 s, and 72°C for 1 min, with a final extension at 72°C for 5 min. First round PCR products from each annealing temperature were unidirectionally sequenced with the ITS4 primer using the Sanger sequencing workflow, as described above.

Following the first round, aliquots of PCR products from three annealing temperatures were combined into one plate; diluted by 2× and a volume of 2 μl was transferred to the second round of PCR to create barcoded libraries with fusion primers containing Ion Xpress™ MID tags and Ion Adapters (Table 2). PCR thermocycling for the second round consisted of 94°C for 2 min, 10 cycles of 94°C for 30 s, annealing at 56°C for 30 s, and 72°C for 1 min, with a final extension at 72°C for 5 min. PCR products were visualized on a 2% agarose gel using an E-Gel96® Pre-cast Agarose Electrophoresis System (Invitrogen, Thermo Fisher Scientific).

In order to amplify the *ITS2* region for *Ginkgo biloba*, the forward primer was designed based on publicly available *Ginkgo* sequences for full *ITS1*, *5*.*8S* and *ITS2* regions (GenBank accessions: EU350117.1, EU643829.1). PCR reactions with ITS-S2F-GINK/ITS4 primers followed the same conditions as described above with the exception that only 56°C was used for annealing in first round of PCR.

PCRClean™ DX kit (Aline Biosciences, Woburn, USA), was used for double size selection purification of amplicons to remove any non-specific amplification products and primer dimers. The beads to product ratio 0.5:1 was used for the upper cut and 0.7:1 –for the lower cut. A volume of 70 μl of product was thoroughly mixed with 35 μl of beads and incubated at room temperature for 9 min, followed 2 min of incubation on DynaMag™-2 magnet (Invitrogen, Thermo Fisher Scientific); 100 μl volume of supernatant was transferred to a tube containing 23.3 μl of water and 29.7 μl of beads for the lower cut, thoroughly mixed by pipetting, incubated for 9 min at room temperature and transferred to the magnet for 2 min or until the solution was clear. The resulting supernatant was discarded and beads were washed three times with 80% ethanol (each time the beads were re-suspended by pipetting and then placed on the magnet for ethanol removal). The beads were dried at room temperature (while sitting on the magnet) until completely dry. Purified PCR products were eluted in 36 μl of water; their concentration was measured on the Qubit 2.0 spectrophotometer using Qubit® dsDNA HS Assay Kit (Invitrogen, Thermo Fisher Scientific). All products were normalized to 1 ng/μl prior to final library dilution (~300×).

Ion PGM™ Template OT2 400 kit was used for template preparation for sequencing as per manufacturer’s protocol except for the recommended library dilutions (we reduced the input of PCR product in library dilution to <12.5 pM). The Ion PGM™ 400 sequencing kit and Ion Torrent PGM™ (Ion Torrent, Thermo Fisher Scientific) were used for sequencing according to manufacturer’s instructions.

NGS data (FASTQ files) were deposited to European Nucleotide Archive (ENA) under the following dataset: http://www.ebi.ac.uk/ena/data/view/PRJEB13560.

### NGS data analysis

Primer sequences were trimmed using Cutadapt (v1.8.1); bases with a quality score less than 20 and reads shorter than 200 bp were removed using Sickle (v1.33); reverse complement was generated using the fast reverse complement function (Fastx Toolkit v0.0.14); resulting reads were clustered into OTUs using Uclust (v1.2.22) with a 2% identity threshold, which was chosen based on reported error rate 1.4–1.5% for Ion Torrent [63–66] and filters with a minimum read depth of 100; OTUs were identified by using a custom ITS database of plant and fungal ITS2 sequences available in Genbank. Each OTU was identified in Qiime (v1.9.0 using BLAST search with a minimum identity of 90% and a minimum e-value of 0.001) using the following command: assign_taxonomy.py -i cluster.fa -m blast -r /path/to/reference/database.fa -t /path/to/reference/taxonomy.txt -e 0.001. Corresponding Qiime scripts are also available at http://qiime.org/scripts/assign_taxonomy.html. To evaluate BLAST-based taxonomic identifications, we calculated the pairwise distance of OTUs generated by Uclust (v.1.2.22) from each supplement to its listed medicinal plant reference sequence in our plant BOLD reference library (boldsystems.org; public dataset: Medicinal Plants—CCDB [DS-RLMPCCDB]), using MEGA ver. 6 [67]. Prior to calculating the distance, all OTUs were aligned against their reference sequence using ClustalW [68], and then checked by eye. All distances were calculated using the Maximum Composite Likelihood model [69] with transitions and transversions included, and uniform rates among sequences. All gaps and indels (insertions and deletions) were excluded; and variance was calculated by using 1000 heuristic bootstrap pseudo-replicates [70]. Distances for each sample were binned into four categories reflecting divergence with the reference sequence. Samples were classified as either identical (0%), low divergence (0–2%), moderate divergence (2–5%), or high divergence (> 5%). Mean pairwise distance for each supplement was also calculated [71] using the same parameters. Taxonomic identification of a cluster was considered robust if it was within 2% of its reference sequence. Any distance beyond 2% was considered a poor taxonomic match.

### *Ginkgo*–chemistry analysis

Samples of Ginkgo products were prepared for HPLC analysis as described in [72] with minor modifications. Ten capsules of each product were combined and pulverized into powder using a mortar and pestle, which were decontaminated with concentrated bleach, DI water, absolute ethanol and UV light before and after each sample. 100 mg of the resulting powder were placed into 20 ml scintillation glass vials; then 20 ml of methanol was added to each sample. Resulting mixtures were sonicated in an ultrasonic bath at 42 kHz at room temperature for 40 min with periodical shaking. A volume of 1 ml was transferred to 1.5 ml tubes and centrifuged for 15 min at 15,000×g; 700 μl of supernatant was applied to Ultrafree MC-GV centrifugal filter units with 0.22 μm Durapore PVDF membrane (EMD Millipore, Merck KGaA, Darmstadt, Germany); and tubes were centrifuged for 2 min at 15,000×g.

Liquid chromatography–mass spectrometry (HPLC-MS) analyses were performed at the Mass Spectrometry Facility of the Advanced Analysis Centre, University of Guelph, using an Agilent 1200 HPLC liquid chromatograph interfaced with an Agilent UHD 6530 Q-Tof mass spectrometer (Agilent Technologies, Santa Clara, USA). A C18 column (Agilent Poroshell 120, EC-C18 50 mm x 3.0 mm 2.7 μm (Agilent Technologies) was used for chromatographic separation with the following solvents: water with 0.1% formic acid (A) and acetonitrile with 0.1%formic acid (B). The mobile phase gradient was as follows: initial conditions were 10% B for 1 min, then increasing to 100% B in 29 min, followed by column wash at 100% B for 5 min and 20 min re-equilibration. The flow rate was maintained at 0.4 ml/min. The mass spectrometer electrospray capillary voltage was maintained at 4.0 kV and the drying gas temperature at 250° C with a flow rate of 8 l/min. Nebulizer pressure was 30 psi and the fragmentor was set to 160 V. Nitrogen was used as both nebulizing and drying gas. The mass-to-charge ratio was scanned across the m/z range of 50–1500 m/z using 2GHz (extended dynamic range) in positive and negative ion modes. The acquisition rate was 2 spectra/s. The instrument was externally calibrated with the ESI TuneMix (Agilent Technologies). The sample injection volume was 10 μl.

Chromatograms were analyzed within Agilent Qualitative Analysis software B 06.0 (Agilent Technologies) finding compounds by the Molecular Feature algorithm using the chemical formulas for quercetin (C_15_H_10_O_7_), quercitrin (C_21_H_20_O_11_), kaempferol (C_15_H_10_O_6_, isorhamnetin (C_16_H_12_O_7_, quercetin-3-beta-glucoside (C_21_H_20_O_12_), rutin (C_27_H_30_O_16_), ginkgolide A (C_20_H_24_O_9_), ginkgolide B (C_20_H_24_O_10_), ginkgolide C (C_20_H_24_O_11_), and bilobalide (C_15_H_18_O_8_); ANOVA with a post-hoc analysis was used to establish pairwise statistically significant differences among the supplements.

## Results and Discussion

### Marker choice for Sanger and Next-Generation Sequencing

Standard markers to be used for authenticating NHP’s have to meet the following criteria: 1) be amplifiable with universal primers; 2) provide good resolution at the species level; 3) have a low instance of homopolymer repeats; and 4) be well represented in available reference sequence databases (NCBI GenBank and BOLD Systems). The length of the marker should not exceed 200–400 bp, in order to allow amplification of degraded DNA and to remain compatible with the read length specifications of the selected NGS platform (e.g., Ion Torrent PGM instrument).

A two-tiered approach was suggested for plant DNA barcoding [19,20]. For the first tier, *rbcL* was selected, which is the best characterized chloroplast gene with sufficient discriminating power, usually to the genus level [19]. It is easy to amplify and sequence; however, the poor discriminatory ability of *rbcL* in closely related species limits its utility in detecting ingredient substitution. Furthermore, the amplified region is 552 bp long, which prohibits its use on older Ion Torrent NGS platforms that until very recently had a read length limit of ~400 bases. For this reason, we only used *rbcL* as an indicator of amplification success in Sanger-based authentication of NHP’s.

We used *ITS2* as a second-tier marker because of its full congruence with the criteria listed above and its prior wide use in plant molecular systematics [73], DNA barcoding [20,60,74,75], and authentication of herbal supplements [11,21]. Region spanning *ITS1*, *5*.*8S* and *ITS2* was also accepted as the standard DNA barcode marker for fungi [76–79]. We targeted the *ITS2* region both for plants and fungi using two forward universal primers: fungal ITS3 and plant ITS_S2F, and ITS4 reverse primer (Table 2). This approach allows estimating overall plant and fungal diversity while giving relatively high resolution for plant species identification.

### DNA degradation vs. PCR inhibition

**Fig 1:**
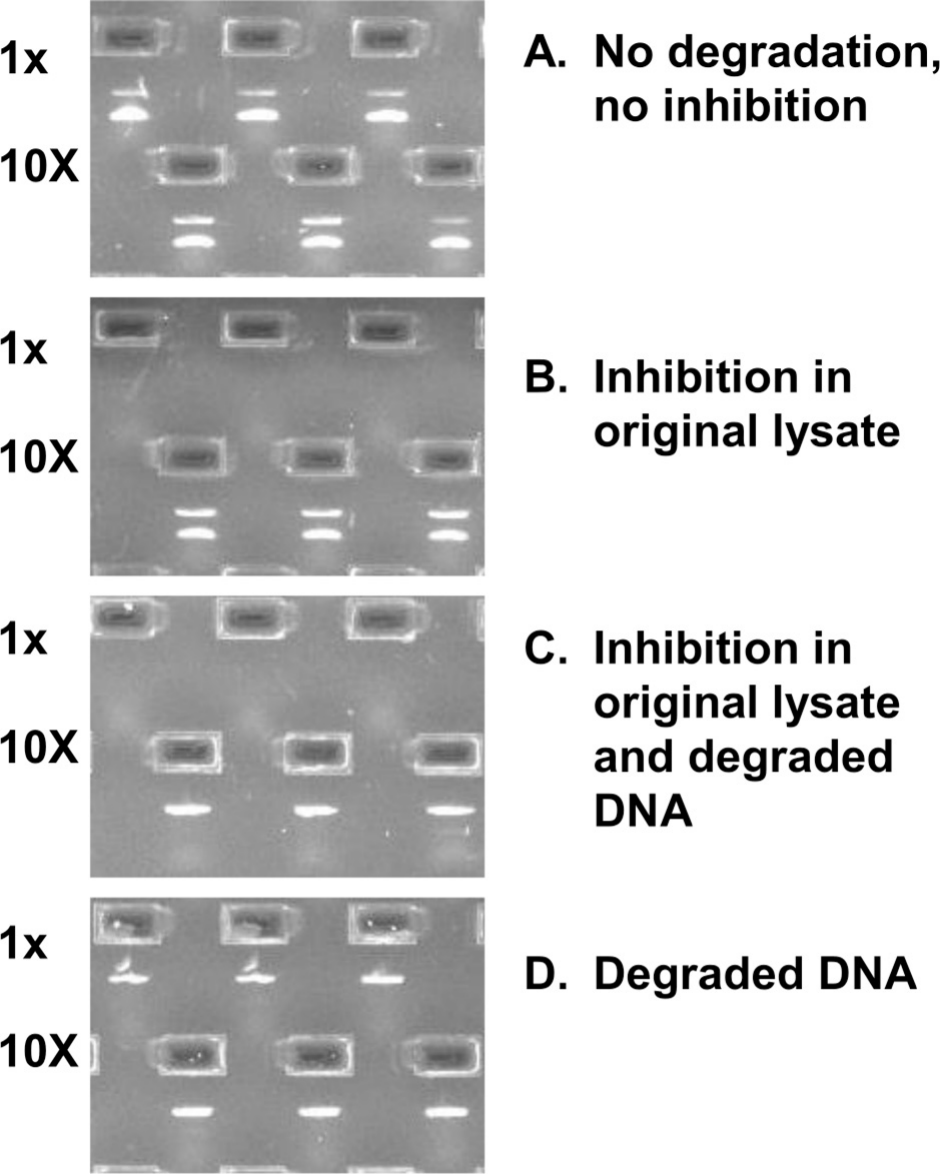
Four scenarios of PCR amplification of short *rbcL* fragment with IAC. (A) Trigonella-13: both amplicons (IAC and target) detected in both lysate dilutions. (B) Echinacea-1: both amplicons (IAC and target) detected only in diluted lysates. (C) Echinacea-3: IAC detected only in diluted lysates. (D) Ginkgo-8: only IAC detected for both lysate dilutions.

DNA extraction from plants is known to be challenging due to the presence of polysaccharides and polyphenolic compounds and despite the availability of a wide selection of commercial kits and taxon-specific methods [80–82], including high-throughput protocols [50,54]. This is especially true for medicinal plants, valued for their secondary metabolites that can complicate extraction and/or inhibit PCR. Because DNA can be degraded or even absent from supplements containing plant extracts, internal positive controls should be used to determine whether failure to recover PCR products is due to the absence of source DNA in the sample, problems with DNA extraction, or the inability to amplify target DNA. One strategy is to add non-target plasmid DNA as an internal amplification control (IAC–see Methods section) to the same sample tube, in order to co-amplify it with the target sequence. Because IAC has the capacity to detect false negative results caused by PCR inhibitors [83–86], it was mandated for PCR-based diagnostic applications [84]. We used the non-competitive strategy [85], when the target DNA and the IAC were amplified by different sets of primers. In our study, we encountered all four possible amplification scenarios (Fig 1) that indicate the importance of IAC in detecting false negative results. We propose using IAC as a standard quality control tool for authenticating herbal supplements.

### Sanger sequencing results

Table 3 summarizes the recovery of DNA from listed and non-listed species as the number of replicates producing non-mixed sequencing signal from at least one direction. Overall, *rbcL* and *ITS2* produced consistent results across all tested buffers for almost each supplement. Reliable detection of source DNA was achieved in only four out of 15 samples (Echinacea-1, Gingko-9, Trigonella-13 and 14). Random detection of source DNA with low sequencing success was observed in Valeriana-4 and 5 (roots) and Hypericum-10 (raw herb). Our results showed preferential amplification of filler DNA (soy) both for *rbcL* and *ITS2* in two standardized extracts (Echinacea-2, Ginkgo-7). They also indicated stochastic preferential PCR amplification of non-listed DNA in many cases where raw herb material could have been contaminated by other plants and fungi (Echinacea-3, Valeriana-4 and 5, Ginkgo-7 and 8, and Hypericum-10). Mixed signal observed in the remaining samples was considered a failure.

**Table 3:** Sanger sequencing results for *rbcL* and *ITS2* using universal primers; numbers refer to successfully sequenced dilution replicates (18 per supplement).

Supplement/Identification	Marker / Buffer / Annealing temperature											
	*rbcL*			*ITS2*								
	CTAB	ILB	WHITL	CTAB			ILB			WHITL		
	55	55	55	50	53	56	50	53	56	50	53	56
**Echinacea-1**												
*Echinacea*	3	3	2	3	3	3	3	3	2	2	2	2
**Echinacea-2**												
*Glycine max*	4	4	6	5	5	5	5	6	6	6	6	6
**Echinacea-3**												
*Daucus carota*	1											
*Epicoccum nigrum*					1							
*Nigrospora*							1					
**Valeriana-4**												
*Valeriana*		2										
*Apieae*		1										
**Valeriana-5**												
*Valeriana*	1											
*Citrus*			1									
*Cladosporium*					1							
*Plantago*				1								
**Valeriana-6**												
**Ginkgo-7**												
*Glycine max*	6	6	4	6	6	6	4	6	5	6	6	6
*Daucus carota*			1									
**Ginkgo-8**												
*Capsicum*		1										
*Aspergillus vitricola*					1							
*Galactomyces*					1							
**Ginkgo-9**												
*Ginkgo biloba*	6	6	6	N/A	N/A	N/A	N/A	N/A	N/A	N/A	N/A	N/A
**Hypericum-10**												
*Hypericum*			3					3				
*Trigonella*	1											
**Hypericum-11**												
**Hypericum-12**												
**Trigonella-13**												
*Trigonella foenum-graecum*				5	6	6	6	6	5	6	6	6
*Trigonella*	6	6	6									
**Trigonella-14**												
*Trigonella foenum-graecum*				6	6	6	6	6	6	6	6	6
*Trigonella*	6	6	6									
**Trigonella-15**												

Simultaneous amplification of multiple DNA sources renders Sanger sequencing results non-interpretable, while the preferential amplification of one or another DNA source results in biased identification outcome. Such results should be interpreted with great caution and indicate a strong need for NGS-based methods.

### NGS: listed medicinal plant and listed filler DNA

**Fig 2:**
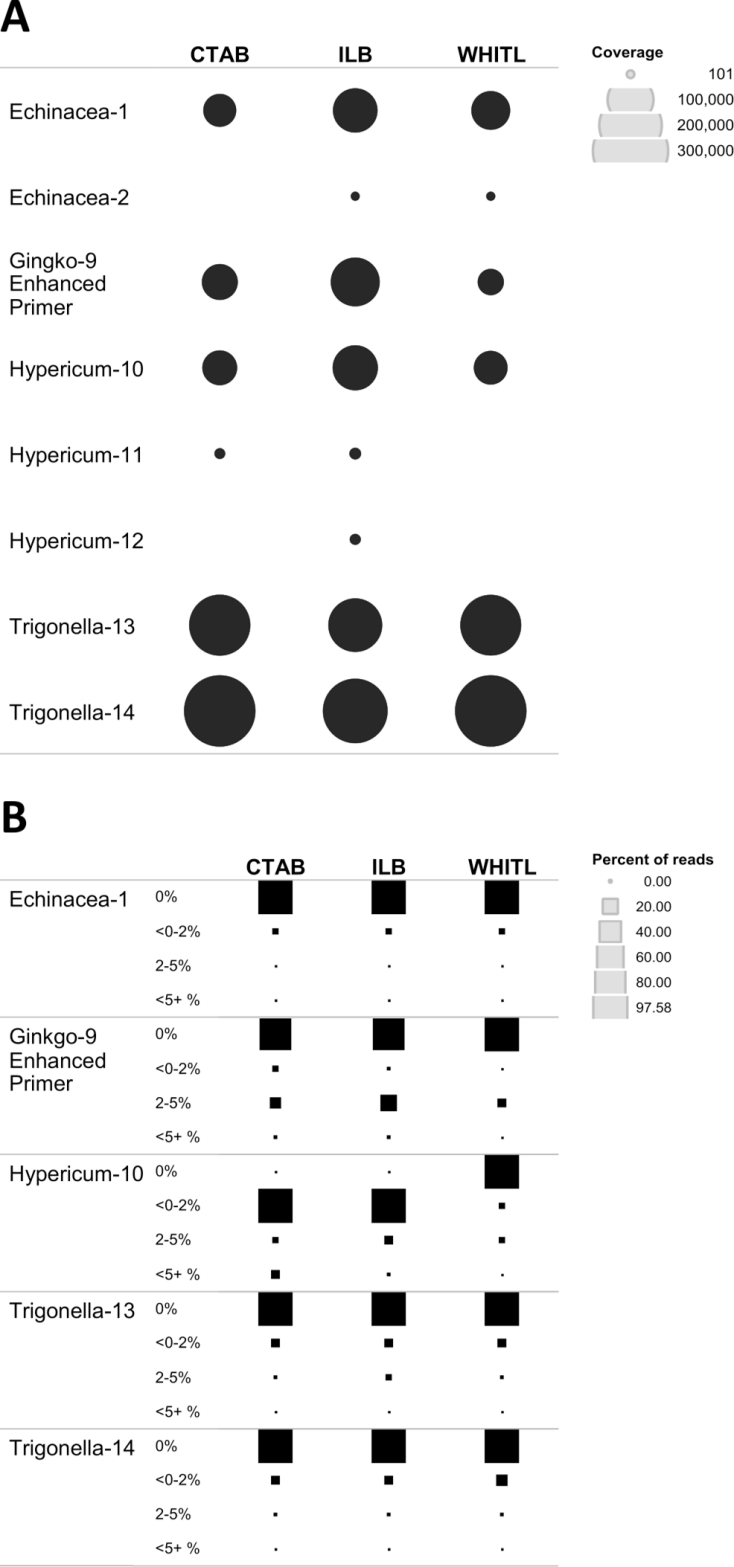
Listed DNA recovery. (A) Summary of target DNA detection with NGS, using custom *ITS* database for plant and fungi downloaded from Genbank. Coverage for *ITS2* is indicated by circle size. (B) Quality of read clusters (OTUs) for five supplements with significant amplification of target DNA. Size of the square reflects percentage of reads falling within a distance threshold of 0%, <0–2%, 2–5%, and <5% from the reference library sequences.

The NGS workflow using the *ITS2* region enabled detection of the key ingredient DNA in 8 out of 15 tested supplements: five raw herb materials and three standardized extracts (Fig 2A). The number of reads for raw herb material (Echinacea-1, Ginkgo-9, Hypericum-10, Trigonella-13 and 14) was markedly higher (21,000–262,500 reads) compared to 135–875 reads for standardized extracts (Echinacea-2, Hypericum-11 and 12). The ILB buffer produced the most consistent results across all preparation forms, especially for standardized extracts.

The quality of read clusters for five supplements with reliable coverage among three lysis buffers was evaluated by comparing the pairwise distance of OTUs from each supplement to its reference sequence in our plant supplement BOLD reference library (Fig 2B). With the exception of Hypericum-10, extracted with CTAB and ILB showing <0–2% distance, the majority of the reads fell into the 0% category, indicating acceptable quality of OTUs used for identification.

Our NGS data provide an insight into Sanger sequencing results. Of the 15 samples analyzed, source DNA was detected in five raw material supplements using both Sanger and NGS (Echinacea-1, Ginkgo-9, Hypericum-10, Trigonella-13 and 14); in three standardized extracts only using NGS (Echinacea-2, Hypericum-11 and 12); and in two raw material supplements (roots) only using Sanger (Valeriana-4 and 5).

The five raw herbal samples that produced high quality *ITS2* OTUs with NGS also generated consistent results with *rbcL* and *ITS2*, confirming that these samples contain high-quality source DNA. Thus, the NGS approach demonstrated reliable capacity to detect source DNA in supplements that failed to produce consistent results with Sanger sequencing (Hypericum-11).

The use of *ITS2* as a reference marker for two of the species studied was hampered by two factors: primer specificity (*Ginkgo biloba*) and intraspecific variability (*Valeriana officinalis*). Our attempt to sequence *ITS2* for *G*. *biloba* using standard *ITS* primers (ITS_S2F and ITS4, Table 2) failed. The design of a new taxon-specific forward primer (ITS-S2F-GINK) solved this problem and resulted in successful authentication of *G*. *biloba* in supplement *Ginkgo*-9, an extract containing dried leaves. Creating the BOLD reference library for *V*. *officinalis* turned out to be the most challenging. Specimens from different locations produced Sanger sequences for *ITS2* with extremely high intraspecific variation (>15%) and the presence of multiple indels. *V*. *officinalis* is known to have variable genome sizes and ploidy levels between different populations varying from 2x to 8x [87,88], suggesting a possible explanation for the sources of the observed intraspecific diversity. Another possible explanation is polymorphism, paralogy, or pseudogenes reported for nrDNA of some angiosperms [89]. As well, our results are concordant with the recent study of Palhares et al. [11], who found similar difficulties while sequencing *Valeriana* root samples. Therefore, use of standard threshold parameters to compare the reads from *Valeriana* supplements with our BOLD reference library was not possible. A potential way to overcome the shortfalls of using *ITS2* in *Valeriana*-containing herbal supplements would be to select a different DNA marker with less intraspecific variability. These examples illustrate that while standard protocols are preferred for authentication of herbal supplements, it may be impossible to provide a universal solution for authentication of all herbal remedies.

Of the five supplements containing listed fillers, both instances of soy filler were confirmed with Sanger and NGS; while only one out of three instances of rice was confirmed with NGS only (Table 1).

### HPLC-MS analysis–*Ginkgo* supplements

**Fig 3:**
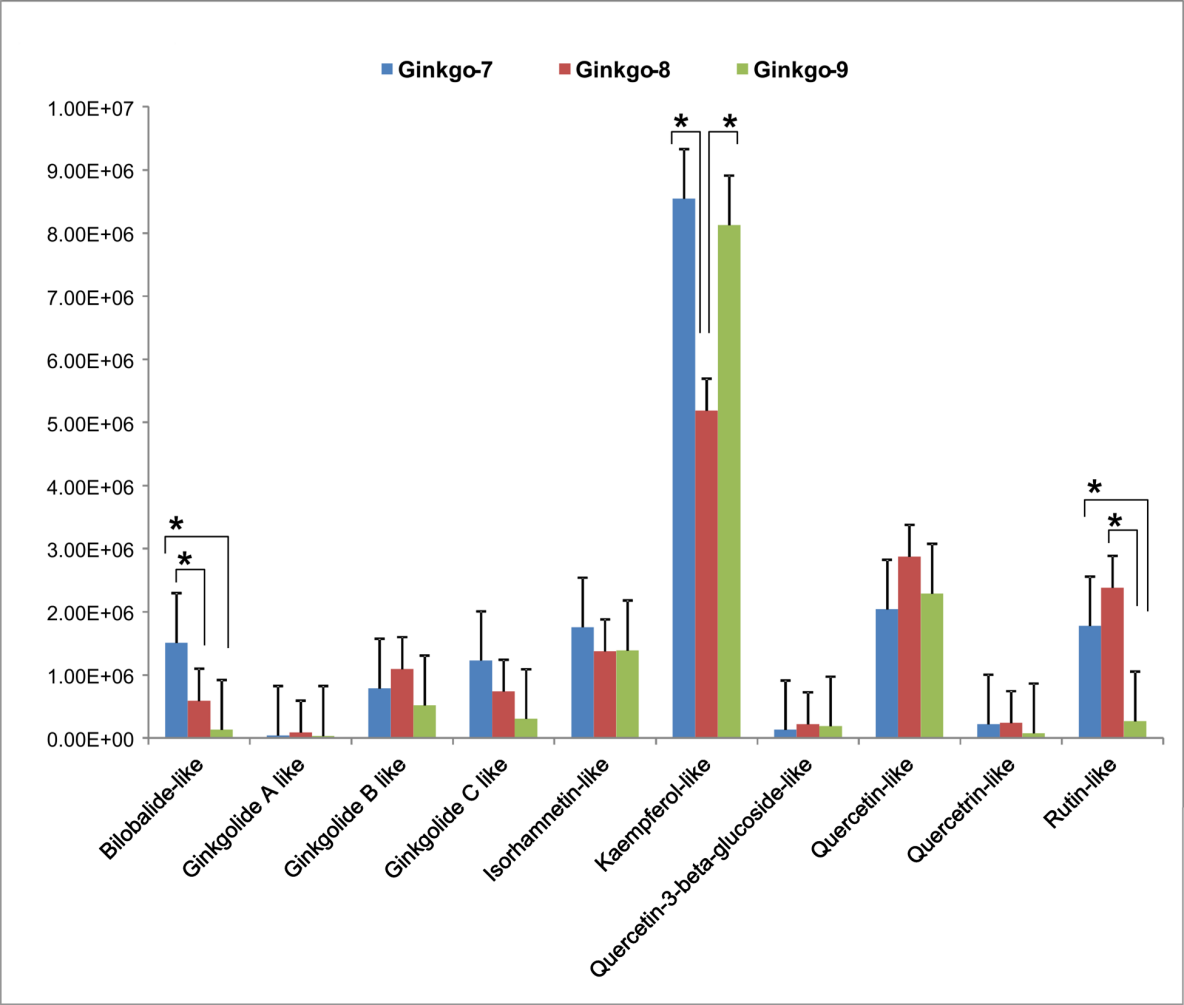
Relative peak height for ten *Ginkgo* active compounds as inferred from Agilent Qualitative Analysis software. Whiskers indicate standard offsets (+SE) of the means; asterisks (*) indicate significant pairwise difference (p<0.05) between mean heights of HPLC-MS peaks for corresponding metabolites.

DNA authentication failed to detect source DNA in all *Ginkgo* supplements containing standardized extracts. In order to verify the presence of active medicinal compounds, all *Ginkgo* supplements were also analyzed with HPLC-MS. All 10 active compounds reported for *Ginkgo* were detected in each of the samples, although their HPLC-MS profiles differed in intensities (Fig 3). Three replicates per each Ginkgo supplement were tested and the resulting values were mostly normally distributed, with very few exceptions. We used one-way ANOVA with a post-hoc analysis to establish pairwise statistically significant differences among the supplements. As the result, only three components for the three supplements showed statistically significant (p<0.05) differences in relative peak heights. Both standardized extracts (Ginkgo-7 and Ginkgo-8) had higher peaks for bilobalide and rutin, while Ginkgo-8 had lower peak for kaempferol.

### NGS: Non-listed plant DNA

**Fig 4:**
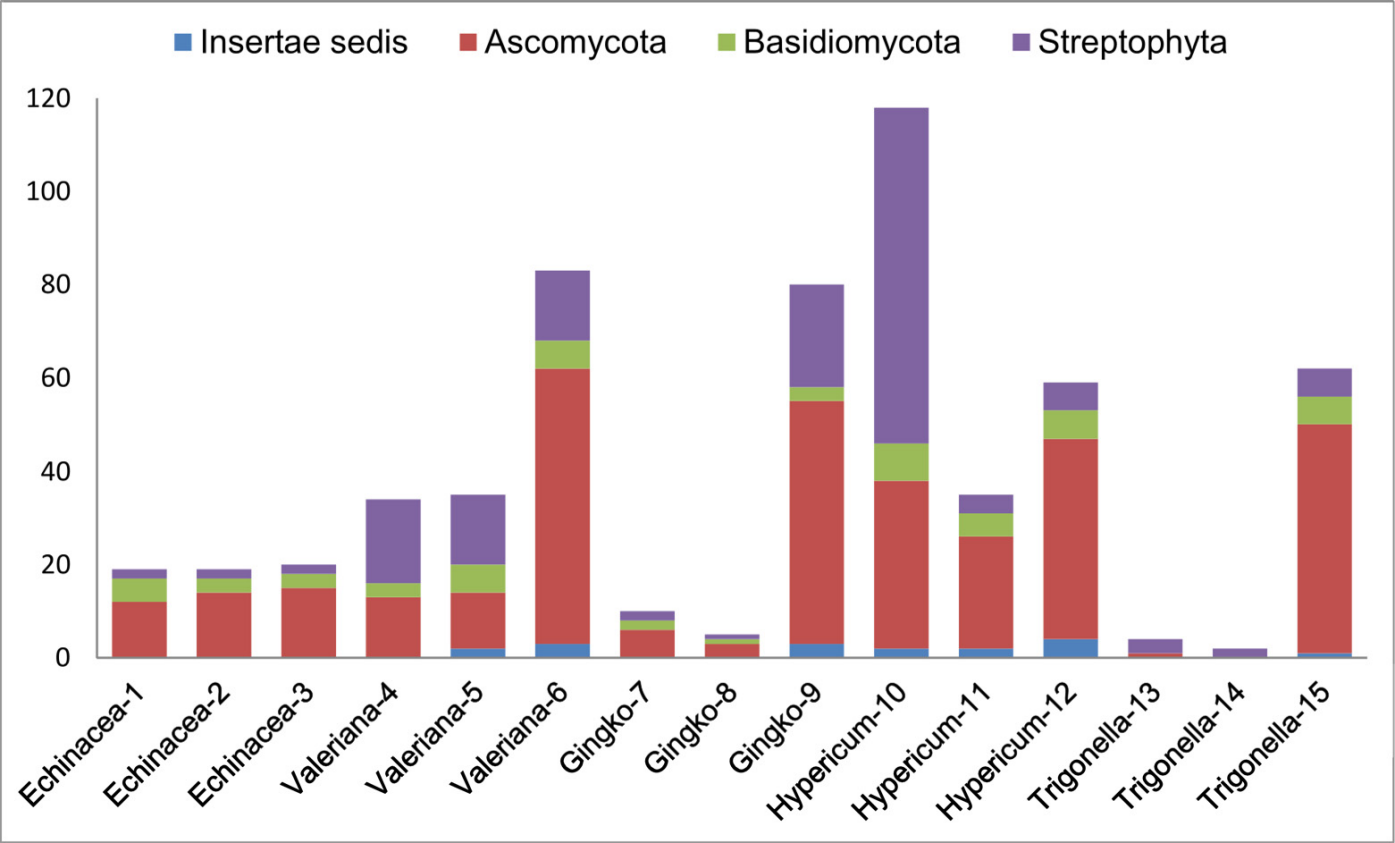
Non-listed, non-filler species count (*ITS2* region with universal primers ITS_S2F/ITS3/ITS4) in herbal supplements tested.

All tested supplements contained non-listed, non-filler plant DNA (Fig 4). The most diverse assembly of plant species was observed in Hypericum-10 supplement, followed by Ginkgo-9 and all *Valeriana* supplements. Overall, the highest number of non-listed, non-filler plant species was detected in raw herb material supplements: roots (*Valeriana*), aerial parts (*Hypericum*), and leaves (*Ginkgo*)–the same samples where target DNA was also detected. All plant extract supplements had lower counts of non-listed plant species. For example, lowest counts were observed in *Ginkgo* extracts (two in Ginkgo-7 and one in Ginkgo-8), thus confirming that isolation of secondary plant metabolites during the manufacturing of standardized extracts leads to DNA degradation or loss. *Trigonella* seed supplements were the only samples that provided a high yield of target DNA, while showing a remarkably low count of non-listed species: three in Trigonella-13 and two in Trigonella-14.

Recent metagenomic studies of clinical samples [90,91] suggest that laboratory background contamination check must be performed with each experiment, so that the contaminants occurring in commercial and in-house prepared reagents could be subtracted from the final results. By running multiple negative controls with all NGS and Sanger sequencing experiments, we detected several common plant and fungal contaminants which were presumed to be in-laboratory contaminants and thus filtered from the final results. The origins of the remaining non-listed plant and fungal DNA cannot be traced with certainty, although we may speculate about the most likely causes.

Many non-listed plants detected in this study are common weed species (mostly from Fabaceae, Asteraceae and Poaceae), suggesting primary trace contamination during bulk harvesting of plant aerial parts. Aside non-selective harvesting, medicinal plants may be ‘naturally’ contaminated with root excretions, sap and pollen from neighbouring plants. Alien pollen can be transferred as a result of inconstant (generalist) foraging by pollinators [92–100]. Root excretions play an important role in pathogen defence mechanisms and are known to contain extracellular DNA [101,102].

Secondary contaminants may be further inadvertently introduced during manufacturing or storage. We detected the presence of other medicinal herbs and species, such as *Matricaria chamomilla*, *Tribulus terrestris*, *Rhodiola crenulata*, *Senna alexandrina*, *Allium spp*., and *Coriandrum sativum*. Plant powders can become airborne or carried over during encapsulation, if the same equipment is used in production of different supplements.

**Fig 5:**
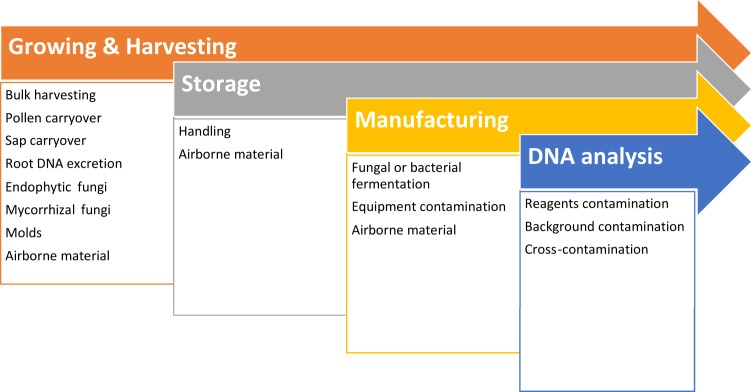
Contamination sources.

To summarize, NGS analysis suggests that, aside from intended or non-intended substitution, possible cross-contamination with trace plant DNA can occur at any stage during growing, harvesting, manufacturing, handling or laboratory analysis of plant material (Fig 5). Detection of such non-target DNA is not always indicative of deliberate adulteration; and such results should be interpreted with caution, especially when legal ramifications are considered.

### Biocomplexity of plant-fungal interactions

NGS analysis detected the presence of fungal DNA in 14 out of 15 tested supplements. The overall fungal count in our study included 111 genera representing 49 families of Ascomycota, followed by 20 genera from 16 families of Basidiomycota and nine genera from 9 families *incertae sedis*. The top five fungal families dominating sequence reads were Pleosporaceae, Nectriaceae, Aspergillaceae, Leptosphaeriaceae and Phaeosphaeriaceae.

The lowest fungal species count was observed in two *Trigonella* seed supplements (one species detected in Trigonella-13 and none in Trigonella-14, Fig 4), which may be explained by antifungal properties of fenugreek [103,104]. Additional two supplements displaying low fungal species counts were standardized *Ginkgo* extracts (Ginkgo-7 and 8), which likely underwent multiple purification stages of active components, in order to remove toxic ginkgolic acids under controlled conditions [27].

The highest fungal species counts were observed in Valeriana-6, Ginkgo-9, and Trigonella-15, followed by all *Hypericum* supplements (Fig 4) which agrees with previous studies on fungal diversity reported from these medicinal plants [105–110].

Fungi and pathogenic bacteria are often found in spices and herbs [111–114]. Tournas *et al*. [115] studied the microbiology of ginseng supplements from the North American market and detected fungal contamination (including potentially toxigenic mold species) in most ginseng supplements, except extracts. A study of incidence and toxigenic capacity of fungal strains (*Aspergillus*, *Penicillium*, and *Fusarium*) in Argentinian medicinal herbs [38] highlighted the need for standard procedures to assess acceptability limits for fungal contamination. Many potentially toxigenic fungi co-exist with plants as important endophytic and/or mycorrhizal symbionts [33–37]. Recent NGS-based studies have reconstructed complex symbiotic network architectures; for example, an assemblage of 33 plant species in a temperate forest in Japan was found to be associated with 387 functionally and phylogenetically diverse fungal taxa [116]. Many endophytic fungi participate in the production of their host’s secondary metabolites and could be used as bio-producers of valuable medicinal components [33,108,117–123]. The production of plant extracts often involves fungal and/or bacterial fermentation to improve yields of bioactive components [8,28,30,31,124]. Diversity of fungi in herbal supplements will be determined by a biocomplexity of plant-fungal interactions, molds proliferated during storage, and strains involved in fermentation, therefore interpretation of test results should focus on potential mycotoxin-producing fungi and human pathogens.

## Conclusions

1. The NGS workflow developed in this study enables simultaneous detection of plant and fungal DNA. This protocol can be utilized by manufacturers for screening of potential mycotoxin-producing and pathogenic fungi, for quality assurance of raw plant materials, contamination control during the production process, and for assessing the purity of the final product.
2. Sanger sequencing should not be used for testing herbal supplements, due to its inability to resolve mixed signal from samples containing multiple species. NGS-based approaches are far more superior, enabling reliable and effective detection of DNA in complex mixtures.
3. Aside from intended or non-intended substitution, cross-contamination with non-target plant DNA may occur at any stage during growing, harvesting, manufacturing, handling or laboratory analysis of plant material. NGS-based methods would detect such traces, in addition to target DNA. By contrast, when the contaminant template is preferentially amplified, Sanger sequencing may detect only contaminant DNA, leading to biased and misleading outcomes.
4. Diversity of fungi in herbal supplements will be determined by a combination of pathogenic, endophytic and mycorrhizal fungi naturally associated with live plant material, saprophytic fungi proliferated during drying and storage, and strains involved in the fermentation during manufacturing of bioactive components. Although this entire spectrum would be easily detected by NGS methods, interpretation of test results should focus on potential mycotoxin-producing fungi and human pathogens.
5. Quality control of herbal supplements should utilize a synergetic approach targeting both bioactive components and DNA, especially for standardized extracts with potentially degraded DNA.

## Supporting Information

S1 Table Plant taxa found in herbal supplements. Read coverage for plant OTUs.
(XLSX)

S2 Table Fungal taxa found in herbal supplements. Read coverage for fungal OTUs.
(XLSX)

S3 Table HPLC-MS analysis of *Ginkgo biloba* supplements. Comparison of ten active components between Ginkgo-7, Ginkgo-8 and Ginkgo-9 supplements.
(XLSX)

## References

[1] Government of Canada HCHP and FBNHPD. Quality of Natural Health Products Guide [Internet]. 2013. Available: http://www.hc-sc.gc.ca/dhp-mps/prodnatur/legislation/docs/eq-paq-eng.php

[2] WHO. The World Medicines Situation 2011—Traditional Medicines: Global Situation, Issues and Challenges [Internet]. WHO Press, Geneva, Switzerland; 2011 Available: http://digicollection.org/hss/en/m/abstract/Js18063en/

[3] Malla S, Hobbs JE, Sogah EK. Functional foods and natural health products regulation in Canada and arouns the world: a summary. In: Canadian Agricultural Innovation Research Network, CAIRN Policy Brief #33 [Internet]. 2013 [cited 3 Dec 2015]. Available: http://www.ag-innovation.usask.ca/cairn_briefs/policybriefs/Mallaetal2final.pdf

[4] Bansal A, Chhabra V, Rawal RK, Sharma S. Chemometrics: A new scenario in herbal drug standardization. J Pharm Anal. 2014;4: 223–233. Available: http://www.sciencedirect.com/science/article/pii/S2095177913001354 29403886 10.1016/j.jpha.2013.12.001 PMC5761221

[5] Steinmann D, Ganzera M. Recent advances on HPLC/MS in medicinal plant analysis. J Pharm Biomed Anal. 2011;55: 744–757. Available: http://www.sciencedirect.com/science/article/pii/S0731708510006588 10.1016/j.jpba.2010.11.015 21131153

[6] Lazarowych NJ, Pekos P. Use of Fingerprinting and marker compounds for identification and standardization of botanical drugs: strategies for applying pharmaceutical HPLC analysis to herbal products. Ther Innov Regul Sci. 1998;32: 497–512. Available: http://dij.sagepub.com/content/32/2/497.short

[7] Poole SK, Dean TA, Oudsema JW, Poole CF. Sample preparation for chromatographic separations: an overview. Anal Chim Acta. 1990;236: 3–42. Available: http://www.sciencedirect.com/science/article/pii/S0003267000832973

[8] Azmir J, Zaidul ISM, Rahman MM, Sharif KM, Mohamed A, Sahena F, et al Techniques for extraction of bioactive compounds from plant materials: A review. J Food Eng. 2013;117: 426–436. Available: http://www.sciencedirect.com/science/article/pii/S0260877413000277

[9] Posadzki P, Watson L, Ernst E. Contamination and adulteration of herbal medicinal products (HMPs): an overview of systematic reviews. Eur J Clin Pharmacol. 2013;69: 295–307. Available: http://www.ncbi.nlm.nih.gov/pubmed/22843016 10.1007/s00228-012-1353-z 22843016

[10] Jiang Y, David B, Tu P, Barbin Y. Recent analytical approaches in quality control of traditional Chinese medicines—a review. Anal Chim Acta. 2010;657: 9–18. Available: http://www.sciencedirect.com/science/article/pii/S0003267009014019 10.1016/j.aca.2009.10.024 19951752

[11] Palhares RM, Gonçalves Drummond M, Dos Santos Alves Figueiredo Brasil B, Pereira Cosenza G, das Graças Lins Brandão M, Oliveira G. Medicinal plants recommended by the world health organization: DNA barcode identification associated with chemical analyses guarantees their quality. PLoS One. 2015;10: e0127866 10.1371/journal.pone.0127866 25978064 PMC4433216

[12] Handy SM, Deeds JR, Ivanova NV, Hebert PDN, Hanner RH, Ormos A, et al A single-laboratory validated method for the generation of DNA Barcodes for the identification of fish for regulatory compliance. J AOAC Int. 2011;94: 201–210. 21391497

[13] Wong EH-K, Hanner RH. DNA barcoding detects market substitution in North American seafood. Food Res Int. 2008;41: 828–837. 10.1016/j.foodres.2008.07.005

[14] Cohen NJ, Deeds JR, Wong ES, Hanner RH, Yancy HF, White KD, et al Public health response to puffer fish (tetrodotoxin) poisoning from mislabeled product. J Food Prot. 2009;72: 810–817. 19435231 10.4315/0362-028x-72.4.810

[15] Hanner R, Becker S, Ivanova N V., Steinke D. FISH-BOL and seafood identification: Geographically dispersed case studies reveal systemic market substitution across Canada. Mitochondrial DNA. 2011;22: 106–122. 10.3109/19401736.2011.588217 21980986

[16] Holmes BH, Steinke D, Ward RD. Identification of shark and ray fins using DNA barcoding. Fish Res. 2009;95: 280–288. 10.1016/j.fishres.2008.09.036

[17] Bottero MT, Dalmasso A. Animal species identification in food products: evolution of biomolecular methods. Vet J. 2011;190: 34–38. 10.1016/j.tvjl.2010.09.024 21041103

[18] Eaton MJ, Meyers GL, Kolokotronis S-O, Leslie MS, Martin AP, Amato G. Barcoding bushmeat: molecular identification of Central African and South American harvested vertebrates. Conserv Genet. 2009;11: 1389–1404. 10.1007/s10592-009-9967-0

[19] CBOL Plant Working Group. A DNA barcode for land plants. Proc Natl Acad Sci U S A. 2009;106: 12794–7. Available: http://www.pnas.org/content/106/31/12794.full 10.1073/pnas.0905845106 19666622 PMC2722355

[20] Hollingsworth PM, Graham SW, Little DP. Choosing and using a plant DNA barcode. PLoS One. Public Library of Science; 2011;6: e19254 Available: http://journals.plos.org/plosone/article?id=10.1371/journal.pone.0019254 10.1371/journal.pone.0019254 21637336 PMC3102656

[21] Newmaster SG, Grguric M, Shanmughanandhan D, Ramalingam S, Ragupathy S. DNA barcoding detects contamination and substitution in North American herbal products. BMC Med. 2013;11: 222 10.1186/1741-7015-11-222 24120035 PMC3851815

[22] Wallace LJ, Boilard SMAL, Eagle SHC, Spall JL, Shokralla S, Hajibabaei M. DNA barcodes for everyday life: Routine authentication of Natural Health Products. Food Res Int. 2012;49: 446–452. Available: http://www.sciencedirect.com/science/article/pii/S096399691200292X

[23] Xin T, Li X, Yao H, Lin Y, Ma X, Cheng R, et al Survey of commercial *Rhodiola* products revealed species diversity and potential safety issues. Sci Rep. 2015;5: 8337 Available: http://www.nature.com/srep/2015/150209/srep08337/full/srep08337.html 10.1038/srep08337 25661009 PMC4321177

[24] Schneiderman ET. Cease and Desist Notification—Herbal Plus—GNC Distributed Herbal Dietary Supplements. In: State of New York Office of the Attorney General [Internet]. 2015 [cited 2 Sep 2015]. Available: http://www.scribd.com/doc/255235564/Cease-and-Desist

[25] Reynaud DTH, Mishler BD, Herbaria J, Neal-kababick J, Brown PN, Health N, et al. The Capabilities and Limitations of DNA Barcoding of Botanical Dietary Supplements [Internet]. 2015 pp. 1–13. Available: http://www.authentechnologies.com/wp-content/uploads/2015/04/Reynaud_DNA_Barcoding_White_Paper.pdf

[26] Novak J, Grausgruber-Gröger S, Lukas B. DNA-based authentication of plant extracts. Food Res Int. 2007;40: 388–392. Available: http://www.sciencedirect.com/science/article/pii/S0963996906001852

[27] Li R, Shen Y, Zhang X, Ma M, Chen B, van Beek TA. Efficient purification of ginkgolic acids from Ginkgo biloba leaves by selective adsorption on Fe_3_O_4_ magnetic nanoparticles. J Nat Prod. 2014;77: 571–575. Available: 10.1021/np400821r 10.1021/np400821r 24484321

[28] Martins S, Teixeira JA, Mussatto SI. Solid-state fermentation as a strategy to improve the bioactive compounds recovery from *Larrea tridentata* leaves. Appl Biochem Biotechnol. 2013;171: 1227–1239. Available: http://www.ncbi.nlm.nih.gov/pubmed/23604970 10.1007/s12010-013-0222-2 23604970

[29] Pandey A. Solid-state fermentation. Biochem Eng J. 2003;13: 81–84. Available: http://www.sciencedirect.com/science/article/pii/S1369703X02001213

[30] Martins S, Mussatto SI, Martínez-Avila G, Montañez-Saenz J, Aguilar CN, Teixeira JA. Bioactive phenolic compounds: production and extraction by solid-state fermentation. A review. Biotechnol Adv. 2011;29: 365–373. Available: http://www.sciencedirect.com/science/article/pii/S0734975011000218 10.1016/j.biotechadv.2011.01.008 21291993

[31] Hölker U, Lenz J. Solid-state fermentation—are there any biotechnological advantages? Curr Opin Microbiol. 2005;8: 301–306. Available: http://www.sciencedirect.com/science/article/pii/S1369527405000457 15939353 10.1016/j.mib.2005.04.006

[32] Southworth D. Defining Complex Interactions Between Plants and Fungi. Biocomplexity of Plant–Fungal Interactions. Wiley-Blackwell; 2012 pp. 205–213. 10.1002/9781118314364.ch10

[33] Kusari S, Hertweck C, Spiteller M. Chemical ecology of endophytic fungi: origins of secondary metabolites. Chem Biol. 2012;19: 792–8. Available: http://www.sciencedirect.com/science/article/pii/S1074552112001998 10.1016/j.chembiol.2012.06.004 22840767

[34] Arnold AE, Mejía LC, Kyllo D, Rojas EI, Maynard Z, Robbins N, et al Fungal endophytes limit pathogen damage in a tropical tree. Proc Natl Acad Sci U S A. 2003;100: 15649–15654. Available: http://www.pnas.org/content/100/26/15649.abstract 14671327 10.1073/pnas.2533483100 PMC307622

[35] Howitz KT, Sinclair DA. Xenohormesis: sensing the chemical cues of other species. Cell. Elsevier; 2008;133: 387–91. Available: http://www.cell.com/article/S0092867408005114/fulltext 10.1016/j.cell.2008.04.019 18455976 PMC2504011

[36] Finlay RD. Ecological aspects of mycorrhizal symbiosis: with special emphasis on the functional diversity of interactions involving the extraradical mycelium. J Exp Bot. 2008;59: 1115–1126. Available: http://jxb.oxfordjournals.org/content/59/5/1115.full 10.1093/jxb/ern059 18349054

[37] Rodriguez RJ, Woodward CJ, Redman RS. Fungal Influence on Plant Tolerance to Stress. Biocomplexity of Plant–Fungal Interactions. Wiley-Blackwell; 2012 pp. 155–163. 10.1002/9781118314364.ch7

[38] Rizzo I, Vedoya G, Maurutto S, Haidukowski M, Varsavsky E. Assessment of toxigenic fungi on Argentinean medicinal herbs. Microbiol Res. 2004;159: 113–20. 10.1016/j.micres.2004.01.013 15293944

[39] Ronaghi M, Uhlén M, Nyrén P. DNA sequencing: a sequencing method based on real-time pyrophosphate. Science (80-). 1998;281: 363–365. 10.1126/science.281.5375.363 9705713

[40] Nyrén P. The history of pyrosequencing. Methods Mol Biol. 2007;373: 1–14. 10.1385/1-59745-377-3:1 17185753

[41] Coghlan ML, Haile J, Houston J, Murray DC, White NE, Moolhuijzen P, et al Deep sequencing of plant and animal DNA contained within traditional Chinese medicines reveals legality issues and health safety concerns. PLoS Genet. 2012;8: e1002657 10.1371/journal.pgen.1002657 22511890 PMC3325194

[42] de Boer HJ, Ichim MC, Newmaster SG. DNA barcoding and pharmacovigilance of herbal medicines. Drug Saf. 2015;38: 611–620. Available: http://www.ncbi.nlm.nih.gov/pubmed/26076652 10.1007/s40264-015-0306-8 26076652

[43] Coutinho Moraes DF, Still DW, Lum MR, Hirsch AM. DNA-based authentication of botanicals and plant-derived dietary supplements: where have we been and where are we going? Planta Med. Georg Thieme Verlag KG; 2015;81: 687–95. Available: https://www.thieme-connect.com/products/ejournals/html/10.1055/s-0035-1545843 10.1055/s-0035-1545843 25856442

[44] Lemmon AR, Emme SA, Lemmon EM. Anchored hybrid enrichment for massively high-throughput phylogenomics. Syst Biol. 2012;61: 727–44. Available: http://sysbio.oxfordjournals.org/content/61/5/727.abstract 10.1093/sysbio/sys049 22605266

[45] Faircloth BC, McCormack JE, Crawford NG, Harvey MG, Brumfield RT, Glenn TC. Ultraconserved elements anchor thousands of genetic markers spanning multiple evolutionary timescales. Syst Biol. 2012;61: 717–26. Available: http://sysbio.oxfordjournals.org/content/61/5/717.long 10.1093/sysbio/sys004 22232343

[46] Parks M, Cronn R, Liston A. Increasing phylogenetic resolution at low taxonomic levels using massively parallel sequencing of chloroplast genomes. BMC Biol. 2009;7: 84 Available: http://www.pubmedcentral.nih.gov/articlerender.fcgi?artid=2793254&tool=pmcentrez&rendertype=abstract 10.1186/1741-7007-7-84 19954512 PMC2793254

[47] Ku C, Hu J-M, Kuo C-H. Complete plastid genome sequence of the basal asterid *Ardisia polysticta* Miq. and comparative analyses of asterid plastid genomes. PLoS One. 2013;8: e62548 Available: http://journals.plos.org/plosone/article?id=10.1371/journal.pone.0062548 10.1371/journal.pone.0062548 23638113 PMC3640096

[48] Ku C, Chung W-C, Chen L-L, Kuo C-H. The complete plastid genome sequence of madagascar periwinkle *Catharanthus roseus* (L.) G. Don: plastid genome evolution, molecular marker identification, and phylogenetic implications in Asterids. PLoS One. 2013;8: e68518 Available: http://journals.plos.org/plosone/article?id=10.1371/journal.pone.0068518 23825699 10.1371/journal.pone.0068518 PMC3688999

[49] Stull GW, Moore MJ, Mandala VS, Douglas NA, Kates H-R, Qi X, et al A targeted enrichment strategy for massively parallel sequencing of angiosperm plastid genomes. Appl Plant Sci. 2013;1 Available: http://www.pubmedcentral.nih.gov/articlerender.fcgi?artid=4105372&tool=pmcentrez&rendertype=abstract 10.3732/apps.1200497 PMC4105372 25202518

[50] Ivanova NV, Fazekas AJ, Hebert PDN. Semi-automated, membrane-based protocol for DNA isolation from plants. Plant Mol Biol Report. 2008;26: 186–198.

[51] Fazekas AJ, Kuzmina ML, Newmaster SG, Hollingsworth PM. DNA Barcoding Methods for Land Plants In: Kress J, Erickson D, editors. Analytical Protocols In: DNA Barcodes, Methods in Molecular Biology New York: Humana Press; 2012 pp. 223–254. 10.1007/978-1-61779-591-6 22684959

[52] Doyle J, Doyle J. A rapid DNA isolation procedure for small quantities of fresh leaf tissue. Phytochem Bull. 1987;19: 11–15.

[53] Ivanova NV, DeWaard JR, Hebert PDN. An inexpensive, automation-friendly protocol for recovering high-quality DNA. Mol Ecol Notes. 2006;6: 998–1002. 10.1111/j.1471-8286.2006.01428.x

[54] Whitlock R, Hipperson H, Mannarelli M, Burke T. A high-throughput protocol for extracting high-purity genomic DNA from plants and animals. Mol Ecol Resour. 2008;8: 736–741. 10.1111/j.1755-0998.2007.02074.x 21585881

[55] Hebert PDN, Penton EH, Burns JM, Janzen DH, Hallwachs W. Ten species in one: DNA barcoding reveals cryptic species in the neotropical skipper butterfly *Astraptes fulgerato*r. Proc Natl Acad Sci U S A. 2004;101: 14812–14817. Available: http://www.pnas.org/content/101/41/14812.long 15465915 10.1073/pnas.0406166101 PMC522015

[56] Hebert PDN, Dewaard JR, Zakharov E V, Prosser SWJ, Sones JE, McKeown JTA, et al A DNA “barcode blitz”: rapid digitization and sequencing of a natural history collection. PLoS One. 2013;8: e68535 10.1371/journal.pone.0068535 23874660 PMC3707885

[57] Levin RA, Wagner WL, Hoch PC, Nepokroeff M, Pires JC, Zimmer EA, et al Family-level relationships of Onagraceae based on chloroplast rbcL and ndhF data. Am J Bot. 2003;90: 107–115. Available: http://www.ncbi.nlm.nih.gov/pubmed/21659085 10.3732/ajb.90.1.107 21659085

[58] Kress WJ, Erickson DL, Jones FA, Swenson NG, Perez R, Sanjur O, et al Plant DNA barcodes and a community phylogeny of a tropical forest dynamics plot in Panama. Proc Natl Acad Sci. 2009;106: 18621–18626. Available: http://www.pubmedcentral.nih.gov/articlerender.fcgi?artid=2763884&tool=pmcentrez&rendertype=abstract 10.1073/pnas.0909820106 19841276 PMC2763884

[59] White T, Bruns T, Lee S, Taylor J. Amplification and direct sequencing of fungal ribosomal RNA genes for phylogenetics In: Innis MA, Gelfand DH, Sninsky JJ, White TJ, editors. PCR Protocols: a guide to methods and applications. Academic Press, New York; 1990 pp. 315–322.

[60] Chen S, Yao H, Han J, Liu C, Song J, Shi L, et al Validation of the ITS2 region as a novel DNA barcode for identifying medicinal plant species. PLoS One. 2010;5: e8613 Available: http://www.pubmedcentral.nih.gov/articlerender.fcgi?artid=2799520&tool=pmcentrez&rendertype=abstract 10.1371/journal.pone.0008613 20062805 PMC2799520

[61] Hajibabaei M, DeWaard JR, Ivanova NV, Ratnasingham S, Dooh RT, Kirk SL, et al Critical factors for assembling a high volume of DNA barcodes. Philos Trans R Soc London B Biol Sci. 2005;360: 1959–1967. Available: //prev200600040765 16214753 10.1098/rstb.2005.1727 PMC1609220

[62] Berry D, Ben Mahfoudh K, Wagner M, Loy A. Barcoded primers used in multiplex amplicon pyrosequencing bias amplification. Appl Environ Microbiol. 2011;77: 7846–9. Available: http://www.pubmedcentral.nih.gov/articlerender.fcgi?artid=3209180&tool=pmcentrez&rendertype=abstract 10.1128/AEM.05220-11 21890669 PMC3209180

[63] Salipante SJ, Kawashima T, Rosenthal C, Hoogestraat DR, Cummings LA, Sengupta DJ, et al Performance comparison of Illumina and Ion Torrent next-generation sequencing platforms for 16S rRNA-based bacterial community profiling. Appl Environ Microbiol. 2014;80: 7583–91. Available: http://www.pubmedcentral.nih.gov/articlerender.fcgi?artid=4249215&tool=pmcentrez&rendertype=abstract 10.1128/AEM.02206-14 25261520 PMC4249215

[64] Lam HYK, Clark MJ, Chen R, Chen R, Natsoulis G, O’Huallachain M, et al Performance comparison of whole-genome sequencing platforms. Nat Biotechnol. 2012;30: 78–82. Available: http://www.pubmedcentral.nih.gov/articlerender.fcgi?artid=4076012&tool=pmcentrez&rendertype=abstract 10.1038/nbt.2065 PMC4076012 22178993

[65] Loman NJ, Misra R V, Dallman TJ, Constantinidou C, Gharbia SE, Wain J, et al Performance comparison of benchtop high-throughput sequencing platforms. Nat Biotechnol. 2012;30: 434–9. Available: 10.1038/nbt.2198 10.1038/nbt.2198 22522955

[66] Quail MA, Smith M, Coupland P, Otto TD, Harris SR, Connor TR, et al A tale of three next generation sequencing platforms: comparison of Ion Torrent, Pacific Biosciences and Illumina MiSeq sequencers. BMC Genomics. 2012;13: 341 Available: http://www.pubmedcentral.nih.gov/articlerender.fcgi?artid=3431227&tool=pmcentrez&rendertype=abstract 10.1186/1471-2164-13-341 22827831 PMC3431227

[67] Tamura K, Stecher G, Peterson D, Filipski A, Kumar S. MEGA6: Molecular Evolutionary Genetics Analysis version 6.0. Mol Biol Evol. 2013;30: 2725–2729. 10.1093/molbev/mst197 24132122 PMC3840312

[68] Larkin MA, Blackshields G, Brown NP, Chenna R, McGettigan PA, McWilliam H, et al Clustal W and Clustal X version 2.0. Bioinformatics. 2007;23: 2947–2948. 10.1093/bioinformatics/btm404 17846036

[69] Tamura K, Nei M, Kumar S. Prospects for inferring very large phylogenies by using the neighbor-joining method. Proc Natl Acad Sci U S A. 2004;101: 11030–11035. 10.1073/pnas.0404206101 15258291 PMC491989

[70] Felsenstein J. Confidence limits on phylogenies: an approach using the bootstrap. Evolution (N Y). 1985;39: 783–791. 10.2307/2408678 28561359

[71] Tamura K, Battistuzzi FU, Billing-Ross P, Murillo O, Filipski A, Kumar S. Estimating divergence times in large molecular phylogenies. Proc Natl Acad Sci U S A. 2012;109: 19333–19338. Available: http://www.pubmedcentral.nih.gov/articlerender.fcgi?artid=3511068&tool=pmcentrez&rendertype=abstract 10.1073/pnas.1213199109 23129628 PMC3511068

[72] Ding S, Dudley E, Plummer S, Tang J, Newton RP, Brenton AG. Quantitative determination of major active components in *Ginkgo biloba* dietary supplements by liquid chromatography/mass spectrometry. Rapid Commun Mass Spectrom. 2006;20: 2753–2760. 10.1002/rcm.2646 16921563

[73] Baldwin BG, Sanderson MJ, Porter JM, Wojciechowski MF, Campbell CS, Donoghue MJ. The ITS region of nuclear ribosomal DNA: a valuable source of evidence on angiosperm phylogeny. Ann Missouri Bot Gard. 1995;82: 247–277. 10.2307/2399880

[74] Chase MW, Cowan RS, Hollingsworth PM, van den Berg C, Madriñán S, Petersen G, et al A proposal for a standardised protocol to barcode all land plants. Taxon. 2007;56: 295–299. Available: http://uoguelph.library.ingentaconnect.com/content/iapt/tax/2007/00000056/00000002/art00004

[75] Kuzmina ML, Johnson KL, Barron HR, Hebert PD. Identification of the vascular plants of Churchill, Manitoba, using a DNA barcode library. BMC Ecol. 2012;12: 25 Available: http://www.biomedcentral.com/1472-6785/12/25 10.1186/1472-6785-12-25 23190419 PMC3538695

[76] Schoch CL, Seifert KA, Huhndorf S, Robert V, Spouge JL, Levesque CA, et al Nuclear ribosomal internal transcribed spacer (ITS) region as a universal DNA barcode marker for Fungi. Proc Natl Acad Sci. 2012;109: 6241–6246. 10.1073/pnas.1117018109 22454494 PMC3341068

[77] Monard C, Gantner S, Stenlid J. Utilizing ITS1 and ITS2 to study environmental fungal diversity using pyrosequencing. FEMS Microbiol Ecol. 2013;84: 165–175. 10.1111/1574-6941.12046 23176677

[78] Ihrmark K, Bödeker ITM, Cruz-Martinez K, Friberg H, Kubartova A, Schenck J, et al New primers to amplify the fungal ITS2 region—evaluation by 454-sequencing of artificial and natural communities. FEMS Microbiol Ecol. 2012;82: 666–677. 10.1111/j.1574-6941.2012.01437.x 22738186

[79] Blaalid R, Kumar S, Nilsson RH, Abarenkov K, Kirk PM, Kauserud H. ITS1 versus ITS2 as DNA metabarcodes for fungi. Mol Ecol Resour. 2013;13: 218–224. 10.1111/1755-0998.12065 23350562

[80] Lim SH, Liew CF, Lim CN, Lee YH, Goh CJ. A simple and efficient method of DNA isolation from orchid species and hybrids. Biol Plant. Kluwer Academic Publishers; 1998;41: 313–316. 10.1023/A:1001863924917

[81] Sweta VP, Parvathy VA, Sheeja TE, Sasikumar B. Isolation and amplification of genomic DNA from barks of *Cinnamomum* spp. Turkish J Biol. 2014;38: 151–155. Available: http://mistug.tubitak.gov.tr/bdyim/abs.php?dergi=biy&rak=1308-5

[82] Focke F, Haase I, Fischer M. DNA-based identification of spices: DNA isolation, whole genome amplification, and polymerase chain reaction. J Agric Food Chem. 2011;59: 513–520. 10.1021/jf103702s 21182302

[83] Rådström P, Löfström C, Lövenklev M, Knutsson R, Wolffs P. Strategies for overcoming PCR inhibition In: Diefenbach CW, Dveksler GS, editors. PCR Primer: A Laboratory Manual. New York: Cold Spring Harbor Laboratory Press; 2003 pp. 149–161. Available: http://cshprotocols.cshlp.org/content/2008/3/pdb.top20.abstract

[84] Hoorfar J, Cook N, Malorny B, Wagner M, De Medici D, Abdulmawjood A, et al Making internal amplification control mandatory for diagnostic PCR. J Clin Microbiol. 2003;41: 5835–5835. Available: http://jcm.asm.org/content/41/12/5835 14662997 10.1128/JCM.41.12.5835.2003 PMC309040

[85] Hoorfar J, Malorny B, Abdulmawjood A, Cook N, Wagner M, Fach P. Practical considerations in design of internal amplification controls for diagnostic PCR assays. J Clin Microbiol. 2004;42: 1863–1868. Available: http://jcm.asm.org/content/42/5/1863 15131141 10.1128/JCM.42.5.1863-1868.2004 PMC404670

[86] Oikonomou I, Halatsi K, Kyriacou A. Selective PCR: a novel internal amplification control strategy for enhanced sensitivity in Salmonella diagnosis. Lett Appl Microbiol. 2008;46: 456–461. 10.1111/j.1472-765X.2008.02340.x 18298457

[87] Hidalgo O, Mathez J, Garcia S, Garnatje T, Pellicer J, Vallès J. Genome size study in the Valerianaceae: first results and new hypotheses. J Bot. 2010;2010: 19 pages. 10.1155/2010/797246

[88] Hidalgo O, Vallès J. First record of a natural hexaploid population for *Valeriana officinalis*: genome size is confirmed to be a suitable indicator of ploidy level in the species. Caryologia. 2012;65: 243–245. Available: http://www.tandfonline.com/doi/full/10.1080/00087114.2012.740193

[89] Bailey C. Characterization of angiosperm nrDNA polymorphism, paralogy, and pseudogenes. Mol Phylogenet Evol. 2003;29: 435–455. Available: http://www.sciencedirect.com/science/article/pii/S1055790303003403 14615185 10.1016/j.ympev.2003.08.021

[90] Salter SJ, Cox MJ, Turek EM, Calus ST, Cookson WO, Moffatt MF, et al Reagent and laboratory contamination can critically impact sequence-based microbiome analyses. BMC Biol. 2014;12: 87 10.1186/s12915-014-0087-z 25387460 PMC4228153

[91] Weiss S, Amir A, Hyde ER, Metcalf JL, Song SJ, Knight R. Tracking down the sources of experimental contamination in microbiome studies. Genome Biol. 2014;15: 564 10.1186/s13059-014-0564-2 25608874 PMC4311479

[92] Brown B, Mitchell R. Competition for pollination: effects of pollen of an invasive plant on seed set of a native congener. Oecologia. 2001;129: 43–49. Available: http://link.springer.com/10.1007/s004420100700 28547066 10.1007/s004420100700

[93] Waser NM. Competition for hummingbird pollination and sequential flowering in two Colorado wildflowers. Ecology. 1978;59: 934–944. 10.2307/1938545

[94] Waser NM. Competition for pollination and floral character differences among sympatric plant species: a review of evidence In: Jones CE, Little RJ, editors. Handbook of experimental pollination biology. Academic Press, New York; 1983 pp. 277–293.

[95] Ganders FR. The biology of heterostyly. New Zeal J Bot. 1979;17: 607–635. Available: http://www.tandfonline.com/doi/abs/10.1080/0028825X.1979.10432574

[96] Schemske DW. Floral convergence and pollinator sharing in two bee-pollinated tropical herbs. Ecology. 1981;62: 946 Available: http://www.esajournals.org/doi/abs/10.2307/1936993

[97] Feinsinger P, Murray KG, Kinsman S, Busby WH. Floral neighborhood and pollination success in four hummingbird-pollinated cloud forest plant species. Ecology. 1986;67: 449 Available: http://www.esajournals.org/doi/abs/10.2307/1938589

[98] Arroyo J, Dafni A. Interspecific pollen transfer among co-occurring heteromorphic and homomorphic species. Isr J Bot. 1992;41: 225–232. 10.1080/0021213X.1992.10677229

[99] Jennersten O, Berg L, Lehman C. Phenological differences in pollinator visitation, pollen deposition and seed set in the sticky catchfly, *Viscaria vulgaris*. J Ecol. 1988;76: 1111–1132. 10.2307/2260638

[100] Rathcke B. Competition and facilitation among plants for pollination In: L. R, editor. Pollination biology. Academic Press, New York; 1983 pp. 305–329.

[101] Hawes MC, Curlango-Rivera G, Wen F, White GJ, Vanetten HD, Xiong Z. Extracellular DNA: the tip of root defenses? Plant Sci. 2011;180: 741–5. 10.1016/j.plantsci.2011.02.007 21497709

[102] Wen F, White GJ, VanEtten HD, Xiong Z, Hawes MC. Extracellular DNA is required for root tip resistance to fungal infection. Plant Physiol. 2009;151: 820–829. 10.1104/pp.109.142067 19700564 PMC2754639

[103] Haouala R, Hawala S, El-Ayeb A, Khanfir R, Boughanmi N. Aqueous and organic extracts of *Trigonella foenum-graecum* L. inhibit the mycelia growth of fungi. J Environ Sci. 2008;20: 1453–1457. Available: http://www.sciencedirect.com/science/article/pii/S1001074208625486 10.1016/s1001-0742(08)62548-6 19209631

[104] Omezzine F, Bouaziz M, Daami-Remadi M, Simmonds MSJ, Haouala R. Chemical composition and antifungal activity of *Trigonella foenum-graecum* L. varied with plant ploidy level and developmental stage. Arab J Chem. 2014; 10.1016/j.arabjc.2014.03.013

[105] El-Nagerabi SAF. Determination of seedborne fungi and detection of aflatoxins in sudanese fenugreek seeds. Phytoparasitica. 2002;30: 61–66. Available: http://link.springer.com/10.1007/BF02983971

[106] Mohamed E, Ghoneem KM. An improved method of seed health testing for detecting the lurked seed- borne fungi of fenugreek. Pakistan J Plant Pathol. 2002; Available: http://agris.fao.org/agris-search/search.do?recordID=PK2002000608

[107] Skórska C, Sitkowska J, Krysińska-Traczyk E, Cholewa G, Dutkiewicz J. Exposure to airborne microorganisms, dust and endotoxin during processing of valerian roots on farms. Ann Agric Environ Med. 2005;12: 119–26. Available: http://www.ncbi.nlm.nih.gov/pubmed/16028876 16028876

[108] Cui Y, Yi D, Bai X, Sun B, Zhao Y, Zhang Y. Ginkgolide B produced endophytic fungus (*Fusarium oxysporum*) isolated from *Ginkgo biloba*. Fitoterapia. 2012;83: 913–20. Available: http://www.sciencedirect.com/science/article/pii/S0367326X12001165 10.1016/j.fitote.2012.04.009 22537641

[109] Rekosz-Burlaga H, Borys M, Goryluk-Salmonowicz A. Cultivable microorganisms inhabiting the aerial parts of *Hypericum perforatum*. Acta Sci Pol—Hortorum Cultus. 2014;13: 117–129.

[110] Zhang H, Ying C, Tang Y. Anti-microbial screening of endophytic fungi from *Hypericum perforatum* Linn. Pak J Pharm Sci. 2014;27: 1153–1156. Available: http://www.ncbi.nlm.nih.gov/pubmed/25176358 25176358

[111] de Boer E, Spiegelenberg WM, Janssen FW. Microbiology of spices and herbs. Antonie Van Leeuwenhoek. 1985;51: 435–438. 10.1007/BF02275058

[112] Hashem M, Alamri S. Contamination of common spices in Saudi Arabia markets with potential mycotoxin-producing fungi. Saudi J Biol Sci. 2010;17: 167–175. 10.1016/j.sjbs.2010.02.011 23961074 PMC3730882

[113] Mandeel QA. Fungal contamination of some imported spices. Mycopathologia. 2005;159: 291–298. 10.1007/s11046-004-5496-z 15770456

[114] Elshafie AE, Al-Rashdi TA, Al-Bahry SN, Bakheit CS. Fungi and aflatoxins associated with spices in the Sultanate of Oman. Mycopathologia. Kluwer Academic Publishers; 2002;155: 155–160. 10.1023/A:1020427527963 12617502

[115] Tournas VH, Katsoudas E, Miracco EJ. Moulds, yeasts and aerobic plate counts in ginseng supplements. Int J Food Microbiol. 2006;108: 178–181. Available: http://www.sciencedirect.com/science/article/pii/S0168160505006100 16434118 10.1016/j.ijfoodmicro.2005.11.009

[116] Toju H, Guimarães PR, Olesen JM, Thompson JN. Assembly of complex plant–fungus networks. Nat Commun. Nature Publishing Group; 2014;5: 5273 Available: http://www.nature.com/ncomms/2014/141020/ncomms6273/full/ncomms6273.html 10.1038/ncomms6273 25327887 PMC4218951

[117] Tan RX, Zou WX. Endophytes: a rich source of functional metabolites. Nat Prod Rep. Royal Society of Chemistry; 2001;18: 448–459. Available: http://pubs.rsc.org/en/Content/ArticleHTML/2001/NP/B100918O 10.1039/b100918o 11548053

[118] Ye Y, Xiao Y, Ma L, Li H, Xie Z, Wang M, et al Flavipin in *Chaetomium globosum* CDW7, an endophytic fungus from *Ginkgo biloba*, contributes to antioxidant activity. Appl Microbiol Biotechnol. 2013;97: 7131–7139. Available: http://www.ncbi.nlm.nih.gov/pubmed/23740314 10.1007/s00253-013-5013-8 23740314

[119] Kusari S, Spitelle M. Metabolomics of Endophytic Fungi Producing Associated Plant Secondary Metabolites: Progress, Challenges and Opportunities. In: Roessner U, editor. Metabolomics. 2012. pp. 241–266. 10.5772/31596

[120] Deshmukh SK, Verekar SA, Bhave S V. Endophytic fungi: a reservoir of antibacterials. Front Microbiol. 2014;5: 715 Available: http://journal.frontiersin.org/article/10.3389/fmicb.2014.00715/abstract 10.3389/fmicb.2014.00715 25620957 PMC4288058

[121] Chandra S. Endophytic fungi: novel sources of anticancer lead molecules. Appl Microbiol Biotechnol. 2012;95: 47–59. Available: http://www.ncbi.nlm.nih.gov/pubmed/22622838 10.1007/s00253-012-4128-7 22622838

[122] Aly AH, Debbab A, Proksch P. Fungal endophytes—secret producers of bioactive plant metabolites. Pharmazie. 2013;68: 499–505. Available: http://www.ncbi.nlm.nih.gov/pubmed/23923629 23923629

[123] Zhao J, Shan T, Mou Y, Zhou L. Plant-derived bioactive compounds produced by endophytic fungi. Mini Rev Med Chem. 2011;11: 159–168. Available: http://www.ncbi.nlm.nih.gov/pubmed/21222580 21222580 10.2174/138955711794519492

[124] Zhou H, Wang C-Z, Ye J-Z, Chen H-X, Tao R, Zhang Y-S. Solid-state fermentation of *Ginkgo biloba* L. residue for optimal production of cellulase, protease and the simultaneous detoxification of *Ginkgo biloba* L. residue using *Candida tropicalis* and *Aspergillus oryzae*. Eur Food Res Technol. 2014;240: 379–388. Available: http://link.springer.com/10.1007/s00217-014-2337-2

