# Correction: Authentication of Herbal Supplements Using Next-Generation Sequencing

**DOI:** 10.1371/journal.pone.0168628

**Published:** 2016-12-13

**Authors:** Natalia V. Ivanova, Maria L. Kuzmina, Thomas W. A. Braukmann, Alex V. Borisenko, Evgeny V. Zakharov

In Table 2, the IonA Forward primer sequence is listed incorrectly. Please see the corrected Table 2 here.

**Table 2:** Primers used for Sanger sequencing, NGS and in IAC.

Region/Primer name	Direction	Primer sequence	Reference
**rbcL**			
rbcLa-F	Forward	ATGTCACCACAAACAGAGACTAAAGC	[57]
rbcLa-R	Reverse	GTAAAATCAAGTCCACCRCG	[58]
MrbcL 163-R1	Reverse	CGGTCCAYACAGYBGTCCAKGTACC	this study
**IAC**			
LepF1	Forward	ATTCAACCAATCATAAAGATATTGG	[55]
LepR1	Reverse	TAAACTTCTGGATGTCCAAAAAATCA	[55]
**ITS2**			
ITS3	Forward	GCATCGATGAAGAACGCAGC	[59]
ITS_S2F	Forward	ATGCGATACTTGGTGTGAAT	[60]
ITS-S2F-GINK	Forward	ATGCGATATTTAGTGTGAAT	this study
ITS4	Reverse	TCCTCCGCTTATTGATATGC	[59]
**NGS-fusion**			
IonA	Forward	CCATCTCATCCCTGCGTGTCTCCGAC [TCAG][IonExpress-MID][**specific sequence**]	Ion Torrent, Thermo Fisher Scientific
trP1	Reverse	CCTCTCTATGGGCAGTCGGTGAT[**specific sequence**]	Ion Torrent, Thermo Fisher Scientific
